# Systematic review of the relation between smokeless tobacco and non-neoplastic oral diseases in Europe and the United States

**DOI:** 10.1186/1472-6831-8-13

**Published:** 2008-05-01

**Authors:** Gerd Kallischnigg, Rolf Weitkunat, Peter N Lee

**Affiliations:** 1 Philip Morris Products S.A., PMI Research & Development, Neuchâtel, Switzerland; 2 P.N. Lee Statistics and Computing Ltd, Surrey, UK

## Abstract

**Background:**

How smokeless tobacco contributes to non-neoplastic oral diseases is unclear. It certainly increases risk of oral mucosal lesions, but reviewers disagree as to other conditions. In some areas, especially South-East Asia, risk is difficult to quantify due to the many products, compositions (including non-tobacco ingredients), and usage practices involved. This review considers studies from Europe (in practice mainly Scandinavia) and from the USA.

**Methods:**

Experimental and epidemiological studies published in 1963–2007 were identified that related risk of oral lesions to smokeless tobacco use. Data were assessed separately for oral mucosal lesions, periodontal and gingival diseases, dental caries and tooth loss, and oral pain.

**Results:**

*Oral mucosal lesions*: Thirty-three epidemiological studies consistently show a strong dose-related effect of current snuff on oral mucosal lesion prevalence. In Scandinavia, users have a near 100% prevalence of a characteristic "snuff-induced lesion", but prevalence of the varied lesions reported in the USA is lower. Associations with chewing tobacco are weaker. The lack of clear association with former use suggests reversibility following cessation, consistent with experimental studies showing rapid lesion regression on quitting.

*Periodontal and gingival diseases*: Two of four studies report a significant association of snuff with attachment loss and four out of eight with gingival recession. Snuff is not clearly related to gingivitis or periodontal diseases. Limited evidence suggests chewing tobacco is unrelated to periodontal or gingival diseases.

*Tooth loss*: Swedish studies show no association with snuff, but one US study reported an association with snuff, and another with chewing tobacco.

*Dental caries*: Evidence from nine studies suggests a possible relationship with use of smokeless tobacco, particularly chewing tobacco, and the risk of dental caries.

*Oral pain*: Limited evidence precludes any clear conclusion.

**Conclusion:**

This review confirms the strong association of current use of smokeless tobacco, particularly snuff, with prevalence of oral mucosal lesions. It provides suggestive evidence of an association of snuff use with gingival recession and attachment loss, and of chewing tobacco with dental caries. While smokeless tobacco clearly increases risk of oral mucosal lesions, interpretation for other endpoints is limited by study weaknesses, including poor confounding control.

## Background

This review concerns epidemiological studies that relate smokeless tobacco [ST] use to non-neoplastic oral diseases and is based primarily on 50 publications, 20 in Scandinavia [1-20], 29 in the USA [21-49] and one in England [50]. In these countries, usage is mainly oral; nasal use of finely ground "dry snuff" has become rare [51]. ST products are traditionally classified as snuff or chewing tobacco (CT) [52]. In the USA, CT is typically mixed with saliva and then placed in the buccal pouch, while finely cut "moist snuff" is frequently held in the lower labial fold. In Scandinavia, snuff (or snus in Sweden) is generally placed under the upper or lower lip. As in some other reviews of ST effects [53-57] results from areas (such as India, South Asia, Africa and Saudi Arabia) where the tobacco chewed is often mixed with other substances, such as betel quid and areca nut are not considered [58]. Nor is oral cancer considered, as its relationship with ST has been discussed elsewhere [57].

An earlier review of non-cancerous and precancerous oral health effects associated with ST use was published by the US Surgeon General in 1986 [53]. This report concluded that "smokeless tobacco is responsible for the development of a portion of oral leukoplakias in both teenage and adult users" and that "dose response effects have been noted by a number of investigators". However, it also noted that "studies of the effects of smokeless tobacco use on gingival and periodontal tissues have resulted in equivocal findings" and that "negative health effects on the teeth from smokeless tobacco use are suspected but unconfirmed". Since that time there have been many reviews of the evidence relating ST use to oral changes [54,55,59-71].

While these reviews are consistent in concluding that ST use is associated with an increased risk of oral mucosal lesions, they are far less consistent with regard to other endpoints. Though various reviews [54,60,61,67] comment on a possible relationship with gingival recession, they disagree on whether an association is clearly demonstrated or not. Similarly, views vary over whether associations with periodontal diseases and dental caries have not been demonstrated [60], are possibly related, based on limited evidence [69], or have been shown to exist [54,67]. A number of these reviews [63,64,68,70,71] note that "ST-related" oral mucosal lesions reverse on quitting and go on to discuss the extent to which the presence of the lesion modifies the risk of oral cancer. A recent review by the US Surgeon General [72] notes that leukoplakia is a classic precursor lesion of oral cancer, but notes that "the leukoplakia that occurs in cigarette smokers differs morphologically from the keratoses caused by smokeless tobacco; although less common, the leukoplakia induced by cigarettes is more susceptible to malignant transformations," citing a review by Bouquot [73]. The importance of distinguishing the oral mucosal lesions induced by ST from oral leukoplakias has also been emphasised by other reviewers [74,75].

Our objective, therefore, was to try to clarify the overall picture by conducting a detailed review of the available evidence.

## Methods

### Study identification and selection

Results were identified from a systematic search of MEDLINE, EMBASE, and SCOPUS through November 2007. The search was not limited by period or language. The main searches were based on combinations of the terms "smokeless tobacco", "chewing tobacco" and "snuff" for exposure, and "oral diseases", "mouth diseases", "periodontal diseases", "leukoplakia", "mucosal lesions", "gingivitis", "gingival recession", "teeth problems", and "dental caries" for outcome. Further articles were identified by inspection of reference lists in individual papers and reviews.

All reports had to satisfy the following *Inclusion criteria*: published in a peer reviewed journal or publicly available; based on research in humans; of experimental, cohort, case-control design or cross-sectional design; study location specified; conducted in North America or Europe; non-neoplastic form of oral disease (ICD 9 520–529) as the outcome; and CT, orally used moist snuff, or unspecified ST as the exposure. They also had to satisfy the *Exclusion criteria*: oral cancer, sample included in a more complete or recent study; inappropriate design (case report, qualitative study or review); and appropriate prevalence estimates or odds ratios (ORs) not reported and cannot be computed from the available data.

### Data extraction

From each report, details were abstracted relating to the study (design, period, region, population, sample selection, and size), the exposure (method of assessment, type of ST investigated, exposure doses and durations considered), the outcome (type of oral disease and method of diagnosis), and issues relating to analysis (type of effect measure, analysis methods, stratification variables, and adjustment factors).

Data were considered separately under four disease groups: oral mucosal lesions, periodontal and gingival diseases, dental caries and tooth loss, and oral pain. Additionally, a summary description of each study was prepared, sorted by study design.

In some studies, the required estimates of prevalence, or of effect size and precision, were not given by the authors and had to be estimated from data presented. Crude ORs and 95% confidence intervals (CIs) were derived from the relevant 2 × 2 table using standard methods, with, where necessary, numbers estimated from proportions given numerically.

## Results

50 principal studies were identified, 20 of snuff, conducted in Scandinavia (two in Denmark, one in Finland and 17 in Sweden), 29 of CT, snuff or overall ST, conducted in the USA, and one of CT, conducted in England. No relevant studies were identified from other parts of North America or Europe. Table 1 (Europe) and Table 2 (USA) give some details of the 50 studies considered. Both tables are sorted by type of study and then by year of publication within type. Six are experimental studies investigating the short-term effect on oral mucosal lesions of quitting ST use, switching to a different brand of snuff, or changing the site of placement of the snuff. Of the 44 epidemiological studies, three are prospective studies and two are case-control studies, though one [5] is a case-control study of oral and oropharyngeal cancer, with only the control group providing data relevant to this report. There are 39 cross-sectional studies, 26 of populations unselected by ST use and 13 of populations selected by ST use and/or presence of oral lesions. Some of the 50 studies are not independent. Thus, two studies [1,19] involve a subset of subjects selected from those in a third [18], while one study [38] gives combined results from a number of groups of baseball players, the results for two of these being previously reported [31,34]. Also, three studies [40,42,43] give results for different endpoints using differently selected samples from the third National Health and Nutrition Examination Survey (NHANES III).

**Table 1:** Details of European studies considered.

**First author, year of publication**	**Location**	**Study Period**^a^	**Sex**	**Age**	**Population**	**Further details of study design**	**Endpoints**^b^
**Experimental studies**							

Larsson 1991 [1]	Sweden (Malmö)	3 to 6 months	M	21 to 70 Mean 36	29 snuff users with degree 2 to 4 mucosal lesions selected from Andersson 1989 [18]	Subjects advised to stop or change their habit	ML
Andersson 1995 [2]	Sweden (Malmö)	12 weeks	Not given	Mean 37	24 users of snuff brand A, 18 users of low-nicotine snuff brand B	Subjects observed for 2 weeks, then brand A users switched to brand B for 10 weeks^c^	ML
Andersson 2003 [3]	Sweden (Malmö)	24 weeks	Not given	Mean 34	20 users of snuff brand A with degree 3 or 4 lesions	Subjects switched to brand B with lower pH for 12 weeks, then to brand C with same pH as brand B but lower nicotine^d^	ML

**Prospective studies**							

Roosaar 2006 [4]	Sweden (Uppsala county)	1973–1974 to 2002	M	15+	1115 men with "snus-induced lesions" in 1973–1974 followed up	Selected from 7890 men examined	ML

**Case-control studies**							

Rosenquist 2005 [5]	Sweden (South)	2001 to 2004	M+F	33 to 87	132 cases of oral and oropharyngeal squamous cell carcinomas, 320 population controls	Cases and controls matched on age ± 3 years, sex and county	ML

**Cross-sectional studies of populations unselected by ST use**							

Tyldesley 1971 [50]	England (Lancashire)	Not given	M	Not given	402 coal miners	-	ML
Modéer 1980 [6]	Sweden (Stockholm)	Not given	M+F	13 to 14 Mean 13.5	232 school children	-	PD
Jungell 1985 [7]	Finland (Tammisaari)	Not given	M	17 to 29	441 military recruits	-	ML
Salonen 1990 [8]	Sweden (Älvsborg county)	1983 To 1984	M^e^	20+	477 randomly selected male adults	-	ML
Hirsch 1991 [9]	Sweden (Gothenburg)	Not given	M+F	14 to 19 Mean 16.8	2145 teenagers attending for dental check-up	-	DC
Wickholm 2004 [10]	Sweden (National)	1985	M+F	31 to 40	1674 adults born on 20th of month	-	PD
Bergström 2006 [11]	Sweden (National)	2002 to 2003	M	26 to 54	84 submariners	-	PD, DC
Montén 2006 [12]	Sweden (Göteborg)	Not given	M	19	103 never smokers	Subsample from larger epidemiological study	PD

**Cross-sectional studies of populations selected by ST use and/or presence of oral lesions**							

Pindborg 1963 [13]	Denmark (Copenhagen)	Not given	M	39 to 83	12 long-term snuff users	SIL probable inclusion criterion	ML
Roed-Petersen 1973 [14]	Denmark (Copenhagen)	1956 to 1970	M+F	< 20 to 90+ Mean 55	450 oral leukoplakia patients	-	ML, OP
Axéll 1976 [15]	Sweden (Not given)	Not given	M	20 to 88 Mean 50	114 snuff users with oral lesions	-	ML
Hirsch 1982 [16]	Sweden (Gothenburg)	Not given	M	15 to 84 Mean 41	50 habitual snuff users	SIL probable inclusion criterion	ML
Frithiof 1983 [17]	Sweden (Stockholm)	Not given	M	31 to 79 Mean 55	21 long-term snuff users referred to dental school for oral lesions	-	ML, PD
Andersson 1989 [18]	Sweden (Malmö)	1986 to 1987	M	17 to 80 Mean 36	252 snuff users; construction and shipyard workers and outpatients	-	ML, PD
Andersson 1994 [19]	Sweden (Malmö)	Not given	M	21 to 75 Mean 42	45 habitual snuff users and 9 users of CT selected from Andersson 1989 [18]	Loose and portion-bag users matched on consumption and usage	ML
Rolandsson 2005 [20]	Sweden (Värmland)	Not given	M	16 to 25 Mean 21	80 ice hockey players, of which 40 were snuff users and 40 non users	Snuff users and non users age matched	ML, PD, DC

^a ^Length of follow-up period for experimental studies, period of follow-up for prospective studies, and time study conducted otherwise

^b ^DC = dental caries, ML = mucosal lesion, OP = oral pain, PD = periodontal or gingival diseases

^c ^Brand A 0.8–0.9% nicotine, 8.2–8.5 pH; Brand B 0.4–0.5% nicotine, 7.8–8.2 pH

^d ^Brand A 0.8% nicotine, 8.6 pH; Brand B 0.8% nicotine, 8.0 pH; Brand C 0.4–0.5% nicotine, 8.0 pH

^e ^Women were also studied but none used snuff

**Table 2:** Details of US studies considered.

**First author, year of publication**	**Location**	**Study Period**^a^	**Sex**	**Age**	**Population**	**Further details of study design**	**Endpoints**^b^
**Experimental studies**							

Grasser 1997 [21]	North Carolina	10 days	M+F	18 to 47	214 soldiers	4 ST users with oral leukoplakia advised to stop	ML
Payne 1998 [22]	Nebraska, Iowa	7 days	M	Mean 25	16 snuff users with oral lesions at habitual sites only	Site of snuff placement changed	ML
Martin 1999 [23]	Texas	6 weeks	M	17 to 34	3051 air force trainees	119 ST users with oral leukoplakia ordered to stop	ML

**Prospective studies**							

Beck 1995 [24]	North Carolina	1988 to 1991	M+F	65+	818 dentate adults (50% black, 50% white)	Selected from Piedmont study. Reexamined at 18 and 36 months	PD, OP
Dietrich 2007 [25]	National	1986 to 2002	M	40–75	43112 health professionals dentate and cancer free at baseline	Data on tooth loss recorded every 2 years	DC

**Case-control studies**							

Fisher 2005A [26]	West Virginia	2001 to 2002	M+F	18+	90 cases of oral leukoplakia, 78 controls with periapical cysts and no oral leukoplakia	Subjects identified from biopsy of hyperkeratosis	ML

**Cross-sectional studies of populations unselected by ST use**							

Greer 1983 [27]	Colorado	Not given	M+F	14 to 19	1119 high school students	-	ML, PD, DC
Poulson 1984 [28]	Colorado	Not given	M+F	14 to 19 Mean 16.7	445 high school students	-	ML, PD, DC
Offenbacher 1985 [29]	Georgia	Not given	M	10 to 17 Mean 13.8	565 grammar and high school students	-	ML, PD, DC
Wolfe 1987 [30]	New Mexico	Not given	M+F	14 to 19 Mean 16.0	226 Native American children at boarding school	-	ML, PD
Cummings 1989 [31]	New York	1985	M	22 to 44 Mean 29	25 baseball players and coaches	-	ML, PD
Creath 1988 [32]	Alabama	Not given	M	11 to 18	995 adolescent football players	-	ML, PD
Stewart 1989 [33]	Florida	Not given	M	10 to 18	114 middle and high school students	5588 M+F interviewed, 182 examined orally, no results for 68F	ML
Ernster 1990 [34]^c^	Arizona	1988	M+F	20 to 29 (77%)	1109 professional baseball players	-	ML, PD, DC
Greene 1992 [35]	Arizona	1989 to 1990	M	20 to 29 (76%)	894 professional baseball players	-	ML
Sinusas 1992 [36]	Pennsylvania	1990	M	17 to 58 Mean 25	206 baseball players and managers	-	ML, PD, DC
Daughety 1994 [37]	Iowa	Not given	M	16 to 17	821 11th and 12th grade school students	-	ML
Robertson 1997 [38]	Arizona	1988 to 1990	M	20 to 29 (77%)	1846 professional baseball players	Includes subjects in Ernster 1990 [34] and Greene 1992 [35]	ML, PD, DC
Tomar 1997 [39]^d^	National	1986 to 1987	M+F	12 to 17	17027 school students	NIDR National Survey of Oral Health	ML
Tomar 1999 [40]	National	1988 to 1994	M+F	18+	14087 dentate adults	Third National Health and Nutrition Examination Survey (NHANES III)	DC
Riley 2004 [41]	Florida	1993 to 2000	M+F	45+	873 adults	Reinterviews occurred over a 48 month period but analyses are all cross-sectional	OP
Shulman 2004 [42]	National	1988 to 1994	M+F	18+	17235 adults	NHANES III, but different endpoints from Tomar 1999 [40]	ML
Fisher 2005B [43]	National	1988 to 1994	M+F	18+	12932 adults	NHANES III, but different endpoints from Tomar 1999 [40] and Shulman 2004 [42]	PD
Sinusas 2006 [44]	Pennsylvania	1991 to 2000	M	Mean 26	190 to 259 baseball players and coaches examined each year	Men attending spring training. Some men may attend on multiple occasions	ML

**Cross-sectional studies of populations selected by ST use and/or presence of oral lesions**							

Smith 1970 [45]	Tennessee	Not given	M+F	Mean 55	15000 long-term snuff users	-	ML
Christen 1979 [46]	Texas	Not given	M	18 to 22 Mean 20	14 college athletes who used ST	-	ML, PD
Kaugars 1992 [47]	Virginia	Not given	M	14 to 77 Mean 29	347 ST users recruited by advertising^e^	-	ML
Little 1992 [48]	Oregon, Washington	Not given	M	15 to 77	245 ST users in Kaiser Permanente Dental Care Program	Also 223 age-matched non ST users	ML
Roberts 1997 [49]	Indiana	1994 to 1995	M+F	Not given	22 snuff users and 19 non users	Oral lesions not recorded in non users	ML

^a ^Length of follow-up period for experimental studies, period of follow-up for prospective studies, and time study conducted otherwise

^b ^DC = dental caries, ML = mucosal lesion, OP = oral pain, PD = periodontal or gingival diseases

^c ^Similar results are reported by Grady *et al *[83], Robertson *et al *[84] and Daniels et al [85]

^d ^Results from this study are also reported by Kleinman *et al *[86]

^e ^An additional group of 91 non users of ST with no oral lesions provides no useful information and has been ignored

The earliest publication [13] was in 1963, with five studies published in the 1970s, 13 in the 1980s, 18 in the 1990s and 13 since 2000. Many of the studies did not give details on when they were conducted. Of the 50 studies, 24 were of males and females, and 24 of males. Two studies [2,3] did not give details on the sex of their subjects. Most of the studies were mainly or wholly of adults, but there were 10 cross-sectional studies of children, mainly of school students, but including one of adolescent football players aged 11 to 18 [32] and one of teenagers attending dental health check-up [9]. Some of the studies of adults involved quite young populations, including air force trainees [23], military recruits [7], college athletes [46], 19 year olds [12] and baseball players [31,34-36,38,44]. Oral mucosal lesions were considered in 40 of the studies, with 19 considering indices of periodontal or gingival diseases, 10 dental caries, and three oral pain.

### Oral mucosal lesions

Fifteen of the studies in Scandinavia provided information relating ST use to oral mucosal lesions. As shown in Table 3, 11 of these used the endpoint "snuff-dipper's lesion" as defined by Axéll *et al *[15] and a further three used an endpoint which appeared to be similar. For convenience we will refer to these 14 studies as being of snuff-induced lesions (SIL). Only in one study [14] was a clearly different endpoint used, of "oral leukoplakia", which could occur both in users and non-users of snuff.

**Table 3:** Definitions of oral mucosal lesions used in studies in Scandinavia

Definition of Axéll 1976 [15]
Snuff dipper's lesion is a lesion of the oral mucosa at the exact site of the regular placing of snuff. The clinical appearance is graded as follows:
Degree 1. A superficial lesion with a colour similar to the surrounding mucosa, and with slight wrinkling. No obvious mucosal thickening.
Degree 2. A superficial, whitish or yellowish lesion with wrinkling. No obvious thickening.
Degree 3. A whitish-yellowish to brown, wrinkled lesion with intervening furrows of normal mucosal colour. Obvious thickening.
Degree 4. A marked, white-yellowish to brown and heavily wrinkled lesion with intervening, deep and reddened furrows and/or a heavy thickening.
Studies using the Axéll 1976 definition
Larsson 1991 [1], Andersson 1995 [2], Andersson 2003 [3], Roosaar 2006 [4], Rosenquist 2005 [5], Axéll 1976 [15], Hirsch 1982 [16], Salonen 1990 [8]^a^, Andersson 1989 [18], Andersson 1994 [19] and Rolandsson 2005 [20]
Studies using other definitions
Jungell 1985 [7] – "snuff-induced lesion", not further defined
Pindborg 1963 [13] – "snuff-induced leukoplakia", characterised by a mucous membrane with "a slightly whitish, sometimes yellowish-brown dry appearance with a very delicately folded or finely grooved appearance"
Roed-Peterson 1973 [14] – "oral leukoplakia", defined as "a white patch not less than 5 mm in diameter which cannot be removed by rubbing, and which cannot be ascribed to any other diagnosable disease"
Frithiof 1983 [17] – "snuff-induced lesion", with "a characteristic whitish appearance frequently with a brown discolouration which clearly contrasted with the neighbouring mucosa"

^a ^32 other types of oral mucosal lesion were also considered, but results were incompletely presented and their incidence (not shown in Table 4) did not appear to be clearly snuff related

Table 4 summarizes the main results from the Scandinavian studies relating oral mucosal lesions to the use of moist snuff (snus). The single study of oral leukoplakia [14], in Denmark, had no control group so could not compare lesion prevalence in snuff users and non-users, though it reported that only 32 of the 450 oral leukoplakia cases studied used snuff and that the presence of symptoms (such as pain or rough feel to the tongue) was much less prevalent in those who used snuff (3.1%) than in those who did not (23.1%, p < 0.01).

**Table 4:** Prevalence of oral mucosal lesions in relation to snuff use evidence from Scandinavia

**Study**	**Location**	**Summary of main results**
**Experimental studies**		
Larsson 1991 [1]	Sweden	Of 29 users with degree 2 to 4 lesions, the lesion disappeared in 20 who quit snuff or changed to portion-bags and changed placement of the quid, reduced in 7 who changed to portion-bags and reduced their exposure, and remained in 2 who modified their habits only slightly.
Andersson 1995 [2]	Sweden	100% lesion prevalence initially in 24 users of ordinary snuff and in 18 users of low nicotine snuff. After 2 weeks, severity was non-significantly lower in habitual users of low nicotine brand. After a further 10 weeks, switching to the low nicotine brand was associated with a reduction in lesion severity (p < 0.01), despite an increased intake of 2.5 g/day.
Andersson 2003 [3]	Sweden	The 20 users of brand A (0.8% nicotine, pH 8.6) had a severity distribution of 0/0/16/4 for degrees 1/2/3/4 respectively. Switching to brand B (0.8% nicotine, pH 8.0) for 12 weeks, reduced the severity to 0/7/13/0, and switching to brand C (0.4–0.5% nicotine, pH 8.0) reduced it further to 2/11/7/0.
**Prospective studies**		
Roosaar 2006 [4]	Sweden	Of 176 users with grade 1–4 lesions in 1973–1974 who were re-examined in 1993–1995, the lesion had disappeared in 62/66 = 94% of those who stopped, and remained in 108/110 = 98% of whose who continued (p < 0.001). Grade 3 and 4 lesions were less common in those who switched to portion-bag snuff, 6/42 = 14%, than in those who continued with loose snus, 20/68 = 29% (0.05 < p < 0.1).
**Case-control studies**		
Rosenquist 2005 [5]	Sweden	100% lesion prevalence in 31 population controls who currently used snuff. Lesion severity was significantly associated with hours/day consumed (p = 0.01), but not with daily consumption (p = 0.07), or duration of use (p = 0.8).
**Cross-sectional studies of populations unselected by ST use**		
Jungell 1985 [7]	Finland	63.6% lesion prevalence in 33 snuff users examined. Of the 12 with no lesions, 8 had quit snuff and 4 started snuff in the previous 3 weeks.
Salonen 1990 [8]	Sweden	92 lesions in 58 snuff only users, 29 in 23 mixed smokers and snuff users, 5 in 235 smokers and 0 in 602 with no tobacco habit (frequencies of subjects with lesion not given).
**Cross-sectional studies of populations selected by ST use and/or presence of lesions**		
Pindborg 1963 [13]	Denmark	100% lesion prevalence in 12 long-term snuff users (lesion prevalence probable inclusion criterion).
Roed-Peterson 1973 [14]	Denmark	Among 450 selected patients with oral leukoplakia, the 32 who used snuff experienced fewer symptoms than the other 418 patients.
Axéll 1976 [15]	Sweden	Among 108 selected snuff users with oral lesions, severity increased with consumption (hours/day or grams/day) of snuff.
Hirsch 1982 [16]	Sweden	100% lesion prevalence in 50 habitual snuff users (lesion prevalence probable inclusion criterion). Severity increased with consumption (hours/day or grams/day) of snuff, and also with duration of use.
Frithiof 1983 [17]	Sweden	Among 21 snuff users referred for treatment due to oral lesions, those who quit following advice to do so had marked lesion improvement in two weeks.
Andersson 1989 [18]	Sweden	100% lesion prevalence in 252 snuff users. Severity was significantly less in 68 users of portion-bag snuff than in 184 users of loose snuff. Severity was also clearly associated with consumption (hours/day or grams/day), though less clearly with duration of use.
Andersson 1994 [19]	Sweden	100% lesion prevalence in 45 snuff users. Severity was less in 23 users of portion-bag snuff than in 22 users of loose snuff.
Rolandsson 2005 [20]	Sweden	Lesion prevalence 87.5% in 40 snuff users and 0.0% in 40 non-users (p < 0.001). Prevalence and severity increased with hours/day of snuff and was lower in users of portion-bag snuff.

The remaining 14 studies generally reported a 100% prevalence of SIL in snuff users. Partly this was because in seven studies [1,3,4,13,15-17] subjects had been, or appeared to have been, selected on the basis of SIL being present, but this was not always the case [2,5,18,19]. The only exceptions were a study in Finland [7] in which all the users with no lesion had either quit or started snuff in the previous three weeks, and a recent study of ice-hockey players in Sweden [20] which reported no lesions in five users of portion-bag snuff. One study in Sweden [8] reported total numbers of lesions, but prevalence seems likely to have been very high in snuff users.

As is evident from the results of the cross-sectional and case-control studies summarized in Table 4, severity of SIL was clearly associated with the length of time per day snuff was used for and with the amount of snuff consumed per day [5,15,16,18,20], though the statistical significance of these relationships could not always be determined. The relationship of severity with duration of use was less marked [5,16,18]. Severity was also lower in users of portion-bag snuff than in users of loose snuff [19,20].

A number of the experimental and epidemiological studies investigated the effect of changing snuff habits on the presence and severity of SIL. The data in Table 4 are all consistent with quitting in the short-term reducing the severity of the lesion [17] and in the longer term eliminating the lesion [1,4,7], and switching to lower nicotine, lower pH or portion-bag snuff also reducing severity [1-4].

While it is clear from the above that SIL occurs in virtually all current users of moist snuff, with severity related to extent of use, the data in Table 4 tell us little about the long-term health implications of the lesion. The study by Roosaar *et al *[4] provides some additional information. Here 1,115 individuals with SIL were followed-up for 27–29 years, with a total of three incident cases of oral cancer seen, two in concomitant daily smokers. Though this was somewhat higher than expected (standardized incidence rate 2.3, 95% CI 0.5–6.7), none of the three occurred at the site of the original SIL.

Of the 15 Scandinavian studies considered, 14 consider use of moist snuff only and not other types of ST. The study of Andersson *et al *[19], however, considered nine users of CT as well as 45 snuff users. They noted that seven of the CT users showed leukoedema in the buccal mucosa, and that six who placed the tobacco on a permanent site had degree 1 or 2 (Axéll) lesions at the site of placement.

The single study in England [50], of coal miners, reported that 10 out of 280 CT users, 3.6%, had leukoplakia, as compared to none out of 122 non-chewers (p = 0.05). Here leukoplakia was defined as "a white patch that could not be rubbed off and which could not be diagnosed as being due to any other disease".

Twenty-four US studies provided evidence relating ST use to oral mucosal lesions. As shown in Table 5, the definitions of the endpoint used varied widely. Eight studies [22,27,28,30,33,36,44,48] used a definition modified by Greer and Poulson [27] from that used by Axéll [15], and at least three studies [34,35,38,39] used a similar definition. However, in some studies the endpoints were clearly not comparable, including one [42] where the endpoint was any type of oral lesion and one [47] where the definition specifically excluded alterations that resolved rapidly on discontinuation of ST use. Unlike the studies in Scandinavia, the conditions were usually referred to as oral leukoplakia or as oral lesions, with no specific reference to ST or snuff in the name of the condition, as in SIL. An exception was the Tomar *et al *study [39] which referred to the endpoint as "smokeless tobacco lesions". Indeed it is clear from Table 6, which presents the prevalence of these lesions in relation to ST use, that the endpoint definitions did not exclude the possibility of the lesion being present in non-users of ST. In fact, in all but one of the 15 studies which presented findings separately for users and non-users, including the Tomar *et al *study, prevalence is greater than zero in the non-users.

**Table 5:** Definitions of oral mucosal lesions used in studies in the USA

Definition of Greer 1983 [27]
Modified version of Axéll 1976 [15]. Oral mucosal lesion graded for severity as:
Degree 1. A superficial lesion with colour similar to that of the surrounding mucosa with slight wrinkling and no obvious thickening.
Degree 2. A superficial whitish or reddish lesion with moderate wrinkling and no obvious thickening.
Degree 3. A red or white lesion with intervening furrows of normal mucosal coour, obvious thickening and wrinkling.
Studies using the Greer 1983 definition
Payne 1998 [22], Greer 1983 [27], Poulson 1984 [28], Wolfe 1987 [30], Stewart 1989 [33], Sinusas 1992 [36], Sinusas 2006 [44] and Little 1992 [48].
Studies using other definitions
Grasser 1997 [21] – oral leukoplakia; a white patch or plaque that does not wipe off and cannot be characterized clinically or pathologically as any other disease
Martin 1999 [23] – oral leukoplakia; modified from Greer 1983 definition, graded for severity as 1: superficial lesion with slight colour change, slight wrinkling and no obvious thickening, 2: superficial white lesion with moderate wrinkling and no obvious thickening, and 3: white lesion with obvious thickening and wrinkling
Fisher 2005 [26] – oral leukoplakia; based on the international classification of diseases, 9^th ^revision code (ICD-9) of 528.6 with a biopsy of hyperkeratosis with or without epithelial atypia or dysplasia. A clinical diagnosis of ST keratosis or frictional keratosis was excluded
Offenbacher 1985 [29] – oral mucosal pathology; alterations considered to represent early changes, as reflected by a mild increase in opalescence and whiteness, with slight furrowing
Creath 1988 [32] – oral leukoplakia; not further defined
Cummings 1989 [31] – soft tissue lesions; not further defined
Ernster 1990 [34] and Greene 1992 [35] – oral leukoplakia; any white, opaque, leathery-appearing plaque not clinically characteristic of another type of white lesion, graded for severity as 1: no or only slight colour change, with or without texture change, 2: colour and texture change, but no thickening, 3: colour and texture change with mild to moderate thickening, or 4: no normal colour, severe texture change, and heavy thickening
Daughety 1994 [37] – oral lesions at placement site; from response to question "Have you ever noticed a sore, white patch or gum problem where you held the tobacco in your mouth?"
Robertson 1997 [38] – oral leukoplakia; any white, opaque, leathery-appearing slightly raised, and irregularly corrugated changes in the oral mucosa not characteristic of another white lesion, graded for severity from 1: slight change in colour and texture to 4: no normal colour, severe texture change and heavy thickening
Tomar 1997 [39] – ST lesions; slight to heavy wrinkling of the mucosa with or without obvious thickening, graded for severity as 1: slight, superficial wrinkling of the mucosa. Colour of the mucosa may range from normal to pale white or grey. Mucosa does not appear to be thickened, 2: distinct whitish, greyish, or occasionally reddish colour change. Wrinkling is obvious, but there is no thickening of the mucosa, or 3: mucosa is obviously thickened, with distinct whitish or greyish colour change. Deep furrows are present within the thickened areas
Shulman 2004 [42] – oral mucosal lesions; 49 types of lesion are listed, including candida-related lesions, tobacco-related lesions, acute conditions and various other conditions, but ST use is only given in relation to the overall incidence of any type
Smith 1970 [45] – oral mucous membrane change "which offered criteria for further study"
Christen 1979 [46] – clinical leukoplakia; a white plaque on the mucosa, with mild to moderate defined as a non palpable, smooth, fairly translucent white area, and severe defined as areas appearing thick, white, indurated and fissured
Kaugars 1992 [47] – oral lesion; a visible alteration of the oral mucosa that persisted for at least 7 days after discontinuation of ST use; an alteration with little probability of resolving within 7 days, in the opinion of the investigator, or an alteration occurring in a subject who was unable to return for a recall visit
Roberts 1997 [49] – oral lesion; any visible lesion

**Table 6:** Prevalence of oral mucosal lesions in relation to ST use – evidence from the USA

			**% prevalence (n)**^a^		
				
**Study**	**Endpoint^b^**	**Exposure^c^**	**Exposed**	**Unexposed**	**Statistical tests and other results and comments^d^**
**Experimental studies**					

Grasser 1977 [21]	Oral leukoplakia	ST ever	13.3 (4)	0.5 (1)	OR 28.2 (3.03–262)
					Lesions in users resolved after 10 days quit
Payne 1988 [22]	Greer 1–3	Snuff current	100.0 (16)^e^	-	After switching site of snuff placement and to reference snuff, all 16 subjects had new lesions after 7 days of use, evident in 15 subjects by day 2. After 7 days, the original lesions at the habitual sites had resolved by 1 degree
Martin 1999 [23]	Oral leukoplakia	ST current	39.4 (119)	1.5 (42)^f^	OR 41.9 (28.6–61.4)
		Snuff current	41.8 (118)	1.5 (42)^f^	OR 46.4 (31.5–68.2)
		CT current	5.0 (1)	1.5 (42)^f^	OR 3.39 (0.44–25.9)
					Lesion had completely resolved in 97.2% (106/109) of ST users 6 weeks after tobacco use was prohibited

**Case-control studies**					

			**Cases**	**Controls**	
Fisher 2005 [26]	Oral leukoplakia	ST current	22.5	7.2	OR 9.21 (1.49–57.0)^g^
		ST former	25.7	12.3	OR 2.73 (0.69–10.8)^g^
		Snuff current	19.0	2.7	OR 30.1 (2.67–338)^g^
		Snuff former	11.1	6.6	OR 0.98 (0.17–5.61)^g^
		CT current	4.2	4.3	OR 0.97 (0.19–4.98)
		CT former	20.0	12.0	OR 1.83 (0.76–4.40)

**Cross-sectional studies of populations unselected by ST use**					

			**Exposed**	**Unexposed**	
Greer 1983 [27]	Greer 1–3	ST use	42.7 (50)	-	Only ST users examined
Poulson 1984 [28]	Greer 1–3	ST use	58.9 (33)	-	Only ST users examined
Offenbacher 1985 [29]	Oral mucosal pathology	ST use	22.7 (17)	4.7 (23)	OR 5.95 (3.00–11.8)
Wolfe 1987 [30]	Greer 1–3	ST current^h^	25.5 (37)	3.7 (3)^f^	OR 8.91 (2.65–29.9)
Creath 1988 [32]	Oral leukoplakia	Snuff ever	5.2 (15)	0.1 (1)	OR 38.6 (5.07–294)
Cummings 1989 [31]	Soft tissue lesion	ST current	17.6 (3)	0.0 (0)	Only 6 never users of ST No differences significant
		ST former	100.0 (2)	0.0 (0)	
		Snuff current	21.4 (3)	0.0 (0)	
		CT current	11.1 (1)	0.0 (0)	
Stewart 1989 [33]	Greer 1–3	ST current	29.0 (9)	-	Prevalence only reported for male current users
Ernster 1990 [34]	Oral leukoplakia	ST current	46.3 (196)	1.4 (7)	OR 59.9 (27.8–129)
		ST former^j^	1.7 (3)	1.4 (7)	OR 1.19 (0.30–4.65)
		Snuff current^k^	58.3 (165)	1.4 (7)	OR 97.1 (44.4–212)
		CT current^k^	17.7 (14)	1.4 (7)	OR 15.0 (5.82–38.4)
Greene 1992 [35]	Oral leukoplakia	ST current	51.7 (167)	2.9	OR 35.8^i^
		ST former^j^	3.5	2.9	OR 1.21^i^
		Snuff current^k^	61.3 (157)	2.9	OR 53.0^i^
		CT current^k^	14.8 (8)	2.9	OR 5.82^i^
Sinusas 1992 [36]	Greer 1–3	ST current	37.1 (23)	6.0 (5)	OR 9.32 (3.29–26.4)
		ST former^l^	6.7 (4)	6.0 (5)	OR 1.13 (0.29–4.39)
		Snuff current^k^	34.2 (13)	6.0 (5)	OR 8.22 (2.67–25.3)
		CT current^k^	16.7 (4)	6.0 (5)	OR 3.16 (0.78–12.9)
Daughety 1994 [37]	Oral lesions at placement site	ST current	33.0 (45)	-	Only ST users asked about oral lesions
		Snuff current	43.9 (25)	-	
Robertson 1997 [38]	Oral leukoplakia	ST current	≈50	< 2	Approximate estimates based on 1846 baseball players in various studies
		ST former	< 2	< 2	
Tomar 1997 [39]	ST lesions	Snuff current	34.9 (107)^m^	1.9 (102)^m^	OR 18.4 (8.5–39.8)^n^
		Snuff former	5.6 (18)^m^	1.9 (102)^m^	OR 2.4 (1.0–6.1)^n^
		CT current	19.6 (54)^m^	3.0 (156)^m^	OR 2.5 (1.3–5.0)^o^
		CT former	6.0 (32)^m^	3.0 (156)^m^	OR 1.3 (0.7–2.2)^o^
Shulman 2004 [42]	Oral mucosal lesions	ST current^p^	60.3 (224)	23.8 (1939)	OR 3.90 (2.75–5.55)^q^
		ST former^p^	12.8 (23)	23.8 (1939)	OR 0.53 (0.25–1.13)^q^
Sinusas 2006 [44]	Greer 1–3	ST current	27.9–46.3^r^	4.0	p < 0.001
		ST former	9.5	4.0	p < 0.001

**Cross-sectional studies of populations selected by ST use and/or presence of oral lesions**					

Smith 1970 [45]	Oral mucous membrane change	Snuff current	11.7 (1751)	-	Only snuff users examined
Christen 1979 [46]	Clinical leukoplakia	ST current	64.3 (9)	-	Only ST users examined
		Snuff current	69.2 (9)	-	
		CT current	57.1 (4)	-	
Kaugars 1992 [47]	Oral lesions	ST current^s^	13.0 (45)	-	Only ST users examined
		Snuff current^s^	14.4 (34)	-	
		CT current^s^	8.4 (18)	-	
Little 1992 [48]	Greer 1–3	ST current	78.8 (193)	6.3 (14)^t^	OR 55.4 (29.8–103)
Roberts 1997 [49]	Oral lesion	Snuff current	31.8 (7)	-	Only snuff users examined

^a ^n = number of subjects with endpoint

^b ^See Table 5 for further definition of endpoint

^c ^Where possible exposure is classified as "current" or "former" with exposure given as "use" only where the source paper did not clearly distinguish how former users were considered. The corresponding non-exposure is "never" or "non-use" to the same type of ST, except where indicated

^d ^ORs are unadjusted for potential confounding variables, except where stated. Where necessary ORs and 95% CIs are calculated from the data provided in the source paper

^e ^Subjects selected to have a lesion at site of snuff placement

^f ^Unexposed group is non-current ST

^g ^Adjusted for age, sex, smoking, alcohol, dental prostheses

^h ^Within the last 7 months

^i ^Numbers of never and former users not available so CI cannot be calculated

^j ^Former includes current in last month but not in last week

^k ^Product usually used

^l ^Former includes those who only used ST in baseball season (study conducted out of season)

^m ^Percentages are based on weighted data; numbers of cases are approximate, calculated by multiplying sample size by weighted percentage

^n ^Adjusted for age, cigarettes, alcohol and CT

^o ^Adjusted for age, cigarettes, alcohol and snuff

^p ^Analyses compare ST users who do not smoke with never users of any tobacco

^q ^Adjusted for age, sex, race and denture use

^r ^Prevalence in current users given only as range over the course of the 10 year study

^s ^Within the past 12 months

^t ^Comparison is with non ST users

Exposure is classified in Table 6 by type (ST, snuff or CT) and by time (current, former, ever or unspecified use which may be equivalent to current). Fourteen of the US studies provided evidence on snuff use. Ignoring one study where the subjects were selected as having a lesion [22], lesion prevalence in current snuff users was typically in the range 30–70%, though lower in two studies [45,47] where the definition implied a more severe lesion, and in one study [31] where the endpoint of "soft tissue lesion" was not further defined. Though lower than the near 100% prevalence reported in Scandinavia, it was clearly much higher than in non-users, with ORs ranging from 8.2 to 97.1 in six studies [23,26,34-36,39]. In contrast the evidence of any increased prevalence in former snuff users is much weaker, with one case-control study [26] reporting no increase, one cross-sectional study [39] an increase of borderline significance, another cross-sectional study [31] an increase, but based on very few subjects, and one experimental study [23] reporting resolution of the great majority of lesions in US Air Force trainees after 6 weeks of a mandatory tobacco-free period.

Nine of the US studies provided evidence on use of CT, and in all of these lesion prevalence was lower in current users of CT than in current users of snuff. Indeed in the five studies where RRs and CIs could be estimated, risk was not elevated (OR 0.97) in one study [26], was elevated but not significantly (ORs 3.39 and 3.16) in two studies [23,36] and was only significantly elevated (ORs 15.0 and 2.5) in two [34,39]. Oral mucosal lesion prevalence was not significantly associated with former CT use [26,39].

Nineteen of the studies provided evidence on overall ST use; in ten of these data by specific type of ST were not available. Given the variation that could have existed in the type of ST used in the different studies (and in the endpoints considered), it is not surprising that there is also marked variation in the prevalence of oral mucosal lesions reported in the different studies. However, it is abundantly clear that there was a marked effect of use on lesion prevalence. Thus, all of the studies for which ORs (with CIs) could be estimated for current or ever ST use showed a significant increase, with ORs over 25 in three studies [21,23,34,48] and the lowest OR, 3.90, being in a study [42] where the endpoint was wide ranging and prevalence relatively high in non-users. Again, the evidence of an association was much weaker for former than for current users. Of the six studies reporting relevant data, five [26,34,36,38,42] found no relationship, with a significant increase in former ST users reported only in one [44].

Aspects of the dose-response relationship have been examined in many of the US studies. In one detailed analysis [34], odds ratios compared to non-users of ST increased significantly with increasing amount of ST used, hours in the month, and recency of last use, with estimates of 354 (129–970) for users of four or more cans of snuff a week, 34.7 (9.4–128) for users of four or more pouches of CT a week, 361 (107–1215) for more than four hours ST use in the day and 201 (84-9-475) for used ST in the last hour. These associations were all found to be independently significant in a multifactorial analysis, but weaker associations with age of initiation and duration of ST use were not independent risk factors. The findings were unaffected by adjustment for age, race, education, cigarette smoking, alcohol consumption, and dental hygiene.

Though not usually analyzed in such detail, similar findings have been reported by other authors. In users of ST or of snuff, prevalence of oral lesions has consistently been found to increase with increasing amount used and/or frequency of use [23,27,28,30,32,35,39,48] and with recency of use [23,35]. These studies also found that prevalence increased with duration of use, though they generally did not test whether the effect of duration remained after adjustment for extent of current usage. Relationships of severity of oral lesions to extent and duration of usage have also been found [23,48], though not always [30].

The epidemiological data from Europe and the USA are consistent with demonstrating a striking and dose-related effect of current snuff use on the prevalence of oral mucosal lesions. In Scandinavia, snuff users have a near 100% prevalence of SIL, but the prevalence of the more varied lesions studied in the USA is typically lower. The association between current CT use and lesion prevalence, mainly studied in the USA, is clearly very much weaker. The lack of any very clear association with former use is consistent with reversibility of the lesion following cessation, and fits in with findings from the experimental studies, which typically show regression of the lesion on quitting after a relatively short period.

### Periodontal and gingival diseases

A variety of indices relevant to periodontal and gingival diseases are considered here, as illustrated in the endpoint column for Tables 7 and 8, where the results relating to ST use are given. Table 7 summarizes findings from seven studies in Sweden, no other Scandinavian studies reporting results, while Table 8 gives results from 13 studies in the USA. Changing and inconsistent diagnostic criteria, as well as a lack of standardized use of methods and terminology [76-81] complicate the summarizing of evidence in this area. The endpoints in Tables 7 and 8 are as named by the authors of the original publications cited, and are defined more precisely there. The results are sorted so that similar endpoints are classified together, in order to facilitate the interpretation of the data. Some of the endpoints considered, such as plaque and calculus, do not of themselves indicate disease, but are indirectly related and the results are shown for additional information.

**Table 7:** Snuff use and endpoints relevant to periodontal and gingival disease – evidence from Sweden

**Study**	**Endpoint**	**Time of exposure**	**Statistic**	**Exposed^a^**	**Unexposed^a^**	**N^b^**	**Summary of statistical tests^c^**
Wickholm 2004 [10]	Plaque index	Ever	% ≥ 2.0	2.6	2.0	4	OR 1.29 (0.45–3.70)^d^
Montén 2006 [12]	Plaque	Current	%	59.0	64.0	19	OR 0.75 (0.32–1.76)
Wickholm 2004 [10]	Calculus index	Ever	% ≥ 2.0	5.9	3.8	9	OR 1.57 (0.76–3.23)^d^
Montén 2006 [12]	Gingivitis	Current	%	47.0	50.0^e^	16	OR 0.94 (0.41–2.15)
Modeer 1980 [6]	Gingival index	Not known	mean	1.10	0.89	-	p < 0.001^f^
Rolandsson 2005 [20]	Gingival index	Current	mean	12.4	13.1	-	Difference not significant
Wickhol m 2004 [10]	Gingival index	Ever	% ≥ 2.0	8.5	12.0	13	OR 0.68 (0.38–1.22)^d^
Rolandsson 2005 [20]	Gingival bleeding	Current	%	10.0	20.0	4	OR 0.44 (0.12–1.62)
Bergström 2006 [11]	Gingival bleeding	Current	-	-	-^e^	-	No significant difference
		Former	-	-	-^e^	-	No significant difference
Frithiof 1983 [17]	Gingival recession	Current	%	9.5	-	2	-
Andersson 1989 [18]	Gingival recession	Current^g^	%	17.8	-	44	Prevalence higher in loose snuff users (23.5%) than in portion-bag users (2.9%), p < 0.05
Wickholm 2004 [10]	Gingival recession	Ever	%	64.7	59.9	99	OR 1.22 (0.87–1.73)^d^
Rolandsson 2005 [20]	Gingival recession	Current	%	17.5	-	-	-
Montén 2006 [12]	Gingival recession	Current	%	42.0	17.0	14	OR 3.72 (1.40–9.99)^h^
Wickholm 2004 [10]	Pocket depth	Ever	%≥ 5 mm	10.5	9.5	16	OR 1.11 (0.64–1.92)^d^
Bergström 2006 [11]	Pocket depth	Current	-	-	-^e^	-	No significant difference
		Former	-	-	-^e^	-	No significant difference
Montén 2006 [12]	Pocket depth	Current^g^	mean	2.3	2.4	-	No significant difference
Montén 2006 [12]	Attachment loss	Current^g^	mean	0.2	0.1	-	No significant difference
Montén 2006 [12]	Alveolar bone level	Current^g^	mean	1.3	1.3	-	No significant difference
Bergström 2006 [11]	Bone Height^j^	Current	mean	1.0	1.06^e^	-	No significant difference^i^
		Former	mean	1.12	1.06^e^	-	No significant difference^i^
Wickholm 2004 [10]	Periodontal disease^l^	Current	-	-	-	-	OR 0.66 (0.30–1.32)^k^
		Former	-	-	-	-	OR 2.55 (0.80–6.80)^k^

^a ^Exposure is always to snuff and is classified, where possible, as current or former. The corresponding unexposed group is never for ever, and non-current for current, except where indicated

^b ^Number of exposed subjects with endpoint (where available)

^c ^Tests are unadjusted for any potential confounding variable, except where stated. Where necessary ORs and 95% CIs are calculated from the data provided in the source paper

^d ^The source paper presented results separately for four groups: A = never used tobacco, B = smoked only, C = snuff only, D = smoked and snuff. The ORs given in Table 7 are based on combining ORs for nonsmokers (C vs A) and smokers (D vs B) using fixed-effects meta-analysis [87], and are thus adjusted for smoking. ORs (CIs) specifically for nonsmokers are plaque index 1.13 (0.14–9.11), calculus index 3.53 (0.93–13.45), gingival index 1.14 (0.39–3.33), gingival recessions 1.43 (0.80–2.55) and pocket depth 1.61 (0.54–4.80)

^e ^Unexposed is never snuff

^f ^Adjusted for plaque index

^g ^Snuff only, no smoking

^h ^The ORs and CIs are adjusted for plaque, gingivitis and toothbrushing

^i ^Adjusted for age

^j ^Distance from the cement-enamel junction to the periodontal bone crest

^k ^Adjusted for smoking and plaque index

^l ^Three or more teeth with pocket depth ≥ 5 mm

**Table 8:** ST use and endpoints relevant to periodontal and gingival disease – evidence from the USA

**Study**	**Endpoint**	**Time of exposure**	**Statistic**	**Exposed^a^**	**Unexposed^a^**	**N^b^**	**Summary of statistical tests^c^**
Ernster 1990 [34]	Plaque	Snuff ever	%	25.8	29.7^d^	154	OR 0.82 (0.65–1.03)
Ernster 1990 [34]	Plaque	CT ever	%	30.2	29.7^d^	52	OR 1.02 (0.72–1.46)
Wolfe 1987 [30]	Calculus	ST current	%^e^	21.6	20.5^f^	-	"Virtually no difference"
Offenbacher 1985 [29]	Gingivitis	ST use	%	72.0	77.1	54	OR 0.76 (0.44–1.32)
Cummings 1989 [31]	Gingivitis	ST current	%	35.3	33.3	6	OR 1.09 (0.15–7.80)
		ST former	%	50.0	33.3	1	OR 2.00 (0.08–51.6)
		CT current	%	33.3	33.3^d^	3	OR 1.00 (0.11–8.95)
		Snuff current	%	28.6	33.3^d^	4	OR 0.80 (0.10–6.25)
Robertson 1997 [38]	Severe gingivitis	ST current	%	-	-	-	"Prevalence equally distributed"
		ST former	%	-	-	-	"Prevalence equally distributed"
Wolfe 1987 [30]	Gingival bleeding	ST current	%^e^	6.2	7.1^f^	-	"Virtually no difference"
Ernster 1990 [34]	Gingival bleeding	Snuff ever	%	5.9	8.8^d^	35	OR 0.64 (0.43–0.96)
Ernster 1990 [34]	Gingival bleeding	CT ever	%	9.9	8.8^d^	17	OR 1.13 (0.65–1.96)
Offenbacher 1985 [29]	Gingival recession	ST use	%	60.0	14.1	45	OR 9.15 (5.40–15.5)^g^
Wolfe 1987 [30]	Gingival recession	ST current	mean %^e^	0.4	0.6^f^	-	"Virtually no difference"
Creath 1988 [32]	Gingival recession	Snuff ever	%	0.3	0.0	1	No significant difference
Cummings 1989 [31]	Gingival recession	ST current	%	41.2	16.7	7	OR 3.50 (0.33–36.9)
		ST former	%	50.0	16.7	1	OR 5.00 (0.15–167)
		CT current	%	55.6	16.7^d^	5	OR 6.25 (0.50–77.5)
		Snuff current	%	35.7	16.7^d^	4	OR 2.78 (0.25–30.9)
Ernster 1990 [34]	Gingival recession	Snuff ever	%	26.4	13.8^d^	158	OR 2.24 (1.73–2.90)^h^
Ernster 1990 [34]	Gingival recession	CT ever	%	11.0	13.8^d^	19	OR 0.77 (0.46–1.29)
Robertson 1997 [38]	Gingival recession	ST use	%	-	-	-	ST users had "significantly more recession"
Christen 1979 [46]	Gingival recession	ST current	%	50.0	-	7	-
Robertson 1997 [38]	Gingival recession increase	ST use	mean (mm)	0.36	No change	-	Not tested
Creath 1988 [32]	Rolled gingival margins	Snuff ever	%	3.1	3.4	9	OR 0.91 (0.42–1.99)
Ernster 1990 [34]	Pocket depth	ST use	% ≥ 4 mm	-	-^d^	-	No significant difference
Robertson 1997 [38]	Pocket depth	ST use	%	-	-	-	No significant difference
Wolfe 1987 [30]	Attachment loss	ST current	%^e^	3.9	3.3^f^	-	"Virtually no difference"
Ernster 1990 [34]	Attachment loss	Snuff ever	%	10.7	4.4^d^	64	OR 2.63 (1.75–3.93)^h^
Ernster 1990 [34]	Attachment loss	CT ever	%	4.7	4.4^d^	8	OR 1.07 (0.49–2.32)
Beck 1995 [24]	Attachment loss (new lesions)^i^	ST use	%	-	-	-	OR 2.99 (p = 0.001)^j^
Beck 1995 [24]	Attachment loss (lesion progression)^k^	ST use	mean	-	-	-	No association^l^
Greer 1983 [27]	Periodontal degeneration^m^	ST use	%	25.6	-	30	-
Poulson 1984 [28]	Periodontal degeneration^m^	ST use	%	26.8	-	15	-
Sinusas 1992 [36]	Periodontal disease^n^	ST use	%	19.3	21.2	17	OR 0.89 (0.42–1.87)
Fisher 2005 [43]	Periodontal disease^o^	ST current	%	9.8	4.3	29	OR 2.1 (1.2–3.7)^p^
Fisher 2005 [43]	Periodontal disease^o^	ST former	%	9.1	4.3	38	OR 1.5 (0.9–2.6)^p^

^a ^Where possible exposure is classified as "current" or "former" with exposure given as "use" only where the source paper did not clearly distinguish how former users were considered. The corresponding non-exposure is never or non-use to the same type of ST, except where indicated

^b ^Number of exposed subjects with endpoint (where available)

^c ^Tests are unadjusted for any potential confounding variable, except where stated. Where necessary ORs and 95% CIs are calculated from the data provided in the source paper

^d ^Unexposed is ST never

^e ^% of sites affected

^f ^Unexposed is ST non-current

^g ^The OR for gingival recession is 20.7 if gingivitis is present and 1.13 if it is not present

^h ^The authors also reported an increase in snuff users after adjustment for age, race, cigarette smoking, alcohol consumption and dental hygiene practice

^i ^During the whole year follow-up period

^j ^Adjusted for income, soft tissue reaction and history of pain

^k ^Increase in depth over a one year period

^l ^ST did not appear as an independent risk factor, following backward elimination, in a logistic regression model involving multiple sociodemographic, psychological, medical, environmental, behavioural and oral variables

^m ^Defined as gingival recession with apical migration of the gingival to or beyond the cementoenamel junction, with or without clinical evidence of inflammation

^n ^Gingival recession or gingival thickening and erythema

^o ^Severe active periodontal disease, defined as having at least one tooth with 6 mm or more attachment loss, and bleeding in the same tooth

^p ^Adjusted for smoking, age, diabetes, minority status, gender and visiting dentist in the past year. Similar estimates of 2.1 (1.0–4.4) for current ST and 1.5 (0.5–4.3) for former ST are given for never smokers, and of 2.1 (1.0–4.2) for current ST and 1.3 (0.7–2.7) for former ST are given for interproximal severe active periodontal disease

The results from Sweden show no significant relationship, in any study, between snuff use and the presence of plaque or calculus, pocket depth, attachment loss, alveolar bone level, bone height or periodontal disease (defined as three or more teeth with pocket depth ≥ 5 mm). One study [6] reported that snuff users had a significantly (p < 0.001) increased gingival index, but other studies [10-12,20] showed no relationship with gingivitis, gingival index or gingival bleeding. Gingival recession was considered by five Swedish studies. Two of these [17,20] provided no data for non-users of snuff, but of the others, one [10] reported the highest rates of recession, but no significant increase in relation to ever snuff use, one [12] reported a significant increase (OR 3.72, 95% CI 1.40–9.99), and one [18] reported a significantly (p < 0.05) higher prevalence in users of loose snuff than portion-bag snuff.

The two US studies to provide some data relating to use of CT [31,34] found no evidence of a relationship with plaque, gingival bleeding or attachment loss, though one of these [31] reported a non-significant increase for gingival recession. However, some of the studies found a relationship between snuff or unspecified ST with some indices of periodontal or gingival diseases. While no significant increases were seen for plaque, calculus, gingivitis, gingival bleeding or pocket depth, the data, though somewhat inconsistent, suggested a relationship with gingival recession. Here, six studies provide relevant data, three in children or adolescents [29,30,32] and three in baseball players [31,34,38]. The two which reported very low rates of gingival recession [30,32] found no association, but four [29,31,34,38] did, significant except in the case of one small study [31]. One of the studies [29] reported high rates of recession and a strong relationship (OR 9.15, 95% CI 5.40–15.5), even stronger in patients with gingivitis. Two studies [24,34] also reported a significant association of snuff or ST with attachment loss, though another study [30] reported no significant relationship. Of two studies of periodontal diseases (see footnotes to Table 8 for definitions), one [36] found no relationship with ST use, but the other [43] reported a significant increase in current users.

Based on the overall data from the US and Sweden, the evidence of a relationship with snuff or unspecified ST use is weak for gingivitis (or gingival bleeding), where a significant association is reported in only one out of eight studies, and for the more general term periodontal disease, where one of the three studies found a relationship. It is, however, rather stronger for attachment loss, where significant associations were reported in two out of four studies, and particularly for gingival recession, where four out of eight studies reported a significant relationship, and an additional study [18] found that prevalence was almost nine times higher in loose snuff users than in portion-bag snuff users. Very limited evidence for CT did not demonstrate any relationship with periodontal or gingival diseases.

### Dental caries

Table 9 (Sweden) and Table 10 (USA) summarize results relating ST use to presence of teeth and indices of dental caries. They are laid out in a style similar to Tables 7 and 8, with results grouped according to the endpoints considered. Endpoints in Tables 9 and 10 are named as in the original publications cited here, with the reader referred to these publications for further definition.

**Table 9:** Snuff use and endpoints relevant to dental caries and tooth loss – evidence from Sweden

**Study**	**Endpoint**	**Time of exposure**	**Statistic**	**Exposed^a^**	**Unexposed^a^**	**Summary of statistical tests^b^**
Rolandsson 2005 [20]	Teeth present	Not known	Mean	27.3	26.9	No significant difference
Bergström 2006 [11]	Teeth present	Current	Median	29	28	No significant difference
Bergström 2006 [11]	Teeth present	Former	Median	28	28	No significant difference
Rolandsson 2005 [20]	Filled teeth	Not known	% any	-	-	OR 1.91 (0.76–4.79)
Rolandsson 2005 [20]	Filled teeth	Not known	Mean	-	-	No significant difference
Hirsch 1991 [9]	Decayed, missing and filled teeth	Not known	Mean	-	-	Increased in users (p < 0.001)
Hirsch 1991 [9]	Decayed proximal surfaces	Not known	Mean	-	-	No significant difference
Hirsch 1991 [9]	Decayed and filled proximal surfaces	Not known	Mean	-	-	Increased in users (p < 0.001)
Hirsch 1991 [9]	Initially decayed proximal surfaces	Not known	Mean	-	-	Increased in users (p < 0.001)

^a ^In the Rolandsson 2005 [20] study, snuff use was compared with snuff non-use; in the Bergström 2006 [11] study, current or former use was compared with never use; in the Hirsch 1991 [9] study, snuff use was compared with no tobacco use

^b ^All statistical tests are unadjusted for any potential confounding variable. Where necessary ORs and 95% CIs are calculated from the data provided in the source paper

**Table 10:** ST use and endpoints relevant to dental caries and tooth loss – evidence from the USA

**Study**	**Endpoint**	**Time of Exposure^a^**	**Statistic**	**Exposed**	**Unexposed^b^**	**Summary of statistical tests^c^**
Robertson 1997 [38]	Teeth present	ST use	Mean	-	-	No significant difference
Tomar 1999 [40]	Teeth present	CT current	Mean	23.89	24.29	No significant difference^d^
Tomar 1999 [40]	Teeth present	Snuff current	Mean	22.99	24.29	p < 0.005^d^
Dietrich 2007 [25]	Tooth loss	CT ever	HR	-	-	HR 1.14 (1.04–1.24)^e^
Greer 1983 [27]	Dental caries	ST use	%	0.0	-	"No evidence of dietary-associated caries"
Poulson 1984 [28]	Dental caries	ST use	%	0.0	-	"Tobacco-associated dental caries ...was absent"
Ernster 1990 [34]	Dental caries	ST use	%	-	-	No significant difference
Sinusas 1992 [36]	Dental caries	ST use	%	7.95	13.56	OR 0.55 (0.19–1.51)
Offenbacher 1985 [29]	Decayed, missing and filled teeth	ST use	Mean	4.05	3.32	0.05 < p < 0.1^f^
Robertson 1997 [38]	Decayed or filled teeth	ST use	Mean	-	-	Higher in users (significance unknown)
Tomar 1999 [40]	Decayed or filled teeth	CT current	Mean	7.99	6.97	p < 0.05^d^
Tomar 1999 [40]	Decayed or filled teeth	Snuff current	Mean	6.11	6.97	No significant difference^d^
Tomar 1999 [40]	Decayed or filled coronal surfaces	CT current	Mean	19.68	17.43	No significant difference^d^
Tomar 1999 [40]	Decayed or filled coronal surfaces	Snuff current	Mean	15.58	17.43	No significant difference^d^
Tomar 1999 [40]	Decayed or filled root surfaces	CT current	Mean	3.84	1.05	p < 0.005^d^
Tomar 1999 [40]	Decayed or filled root surfaces	Snuff current	Mean	0.86	1.05	No significant difference^d^
Tomar 1999 [40]	Decayed or filled root surfaces	CT current	%	-	-	OR 4.18 (1.96–8.92)^g^
Tomar 1999 [40]	Decayed or filled root surfaces	Snuff current	%	-	-	OR 0.67 (0.26–1.74)^g^
Tomar 1999 [40]	Decayed root surfaces	CT current	Mean	3.24	0.88	p < 0.005^d^
Tomar 1999 [40]	Decayed root surfaces	Snuff current	Mean	0.81	0.88	No significant difference

^a ^In all studies except two, exposure is ST use unspecified as to whether current or ever. In Tomar 1999 [40] exposure is either current CT and no other form of tobacco, or current snuff and no other form of tobacco, and in Dietrich 2007 [25] exposure ever CT

^b ^In all studies except two, comparison is with ST non-use. In Tomar 1999 [40] it is with never tobacco and in Dietrich 2007 [25] it is with never CT

^c ^All statistical tests are unadjusted for any potential confounding variable, unless otherwise indicated. Where necessary ORs and 95% CIs are calculated from the data provided in the source paper

^d ^Adjusted for age, and race or ethnicity

^e ^Hazard ratio (HR) adjusted for age, other tobacco use, race, BMI, physical activity, diabetes, profession, routine medical examination, alcohol, calorie intake, multivitamin use and Vitamin C supplement use

^f ^Estimated from means and standard errors given separately for subjects with and without gingivitis, between which groups no significant differences were seen associated with ST use

^g ^Adjusted for age, race or ethnicity, education and past-year dental visit

Two studies from Sweden [11,20] provided no indication of an effect of snuff use on the number of teeth present. One of these studies [20] also reported no relationship with having filled teeth. The third study [9], of 14–19 year olds attending dental check-up, reported significant (p < 0.001) increases in snuff users in the mean number of decayed, missing and filled teeth, of decayed and filled proximal surfaces and of initially decayed proximal surfaces, though not in the mean number of decayed proximal surfaces. It should be noted, however, that these analyses were not adjusted for age, the snuff dippers being markedly older (71% aged 17–19) than the non-tobacco users (52% aged 17–19, p < 0.001). Within specific years of age, significant differences were generally not seen (except for decayed, missing and filled teeth in 17 year olds), but an overall age-adjusted test was unfortunately not reported. The analyses were also not adjusted for education.

Tooth loss in relation to ST use was investigated in three US studies. While one [38] found no association with ST use, one [40] found a significant relationship with current snuff but not current CT use, and the third [25] reported a significant 14% increase in the rate of tooth loss in a large prospective study in which adjustment was made for a range of potential confounding variables, including age and social status.

Dental caries was studied in seven US studies. In six of these [27-29,34,38,44] there was no significant evidence of a relationship with unspecified ST use. The seventh and by far the most comprehensive investigation [40] was based on the NHANES III study. Results for a range of the endpoints studied are shown in Table 10. No significant difference was seen for snuff use for any index of dental caries (though, as noted above, it was for number of teeth present). In contrast, CT use was significantly associated with decayed or filled permanent teeth, decayed or filled root surfaces, and decayed root surfaces. In an analysis adjusted for age, race/ethnicity, education, and past-year dental visits, the OR for decayed or filled root surfaces was 4.18 (1.96–9.92) for current users of CT only and 0.67 (0.26–1.74) for current users of snuff only, compared to never users of tobacco. In current users of CT, the OR increased with the number of packs per week (trend p = 0.0002) and with years of use (trend p = 0.002). None of the other studies reported dose-response data for dental caries.

The overall evidence is suggestive of a possible relationship of ST use, particularly CT, with the risk of dental caries.

### Oral pain

Information relating ST use to oral pain is extremely limited. In the Florida Dental Care Study [41], 33 adults aged 45+ were current users of CT or snuff, 69 were former users, and 604 had never been users. Based on reports over a 48 month period, analyses were carried out relating current and former ST use to five pain variables (tooth pain, painful gums, temperature sensitivity, activity reduction, and trouble sleeping because of oral pain), with adjustment for race, sex, age, oral hygiene, dental care, and education. Former use was unrelated to any of the five pain variables. For current use, a significant increase was noted for painful gums (OR 1.7, 95% CI 1.2–2.1), but not for the other four variables, with the odds ratio for tooth pain being 1.0 (95% CI 0.4–2.3). Separate results for CT and for snuff were not presented.

No other study directly related ST to the prevalence of oral pain, but a study in Denmark [14] found that among oral leukoplakia patients, snuff users were less likely to experience pain from the lesion than were non-snuff users (1/32 = 3.1% vs 83/399 = 20.8%, p < 0.01). The North Carolina study [24] reported that ST use and history of pain both predicted new cases of attachment loss over a three year period but did not relate ST use to pain.

It is not possible to draw any clear conclusion on the relationship between ST use and oral pain from these data.

## Discussion

This report is based on data from 50 studies published between 1963 and 2007 relating the risk of non-neoplastic oral diseases to the consumption of CT and snuff as used in Europe (mainly Scandinavia) and the USA. Six were experimental studies (or had an experimental component); the epidemiological studies were mainly of cross-sectional design. The number of studies considered is substantially larger than in other recent reviews (e.g. [66-71]). Many of the 50 reports have limitations and present less information than is ideal. Problems encountered include small numbers of subjects or exposed cases, unrepresentativeness of the studied populations, inconsistently defined outcomes, and heterogeneous methods of exposure assessment. Exposure details such as type of ST and duration and frequency of use were often not reported. A number of the studies only presented data on populations selected by ST use and/or presence of oral lesions, and therefore, did not allow estimation of prevalences and odds ratios. At the analytical level, one major weakness was incomplete presentation of findings. Another was failure to adjust for important potential confounders, with only a handful of studies adjusting for variables such as age, smoking, education or frequency of dental visits. A third was the frequent failure to present results separately for major subgroups, particularly regarding alternative tobacco consumption, either smoked or smokeless. Thus comparisons of ever snuff users with never snuff users may also compare groups differing on the use of cigarettes and other ST. These shortcomings inevitably limit the inferences that can be drawn as well as the possibility to conduct meaningful meta-analyses. Nevertheless, we feel that it is possible to draw some conclusions from the present results.

It is abundantly clear, as numerous reviewers agree [53-55,59-71], that snuff use markedly increases the risk of oral mucosal lesions. In Scandinavia, users have a near 100% prevalence of the characteristic SIL, a lesion which appears not to occur in non-users, though direct evidence from the available publications is limited to one study [20]. In the USA, the types of lesion studied are much more varied. There many snuff users do not have a lesion and some non-users do, but prevalence is much higher in users than in non-users, with reported odds ratios of up to almost 100 [34], and frequency dose-related to daily usage. Though strong dose-related relationships are also clearly seen in relation to unspecified ST use, the association between current CT use and lesion prevalence, mainly studied in the USA, is clearly very much weaker.

In both the USA and Scandinavia prevalence of oral mucosal lesions in former users of snuff, CT or unspecified ST is typically low and not clearly higher than in never users, suggesting the reversibility of the effect. This is consistent with the findings from experimental studies where rapid regression upon quitting was observed as well as rapid onset in subjects starting to use ST in the first place or switching to a new habitual oral site. The experimental studies also suggest that the severity (or even the appearance) of the lesion can be affected by switching the type of product used.

The rapid reversibility on quitting ST of SIL, and many of the oral mucosal lesions studied in the USA, suggests that presence of these lesions may not be of great clinical significance. Additional evidence that there is a low probability of SIL transforming into oral cancer comes from the recent follow-up study of Roosaar *et al *[4] which found no cases of oral cancers at the site of snuff placement in over 1000 individuals with SIL followed for over 25 years, the low incidence of oral cancer in Sweden, where use of oral snuff is very common [64], and evidence that the lesions induced may be less susceptible to malignant transformation than those induced by cigarette smoke [73]. While it is well documented that cigarette smoking increases the risk of oral cancer [72,82], a recent review of ST as currently used in Western populations suggests that it carries little or no increased risk of oral cancer [57].

The evidence available for oral pain is too limited to draw a clear conclusion regarding an association. Somewhat more evidence is available (see Tables 5 to 8) regarding periodontal diseases, caries and related conditions. Although one study [6] reported a significantly higher prevalence of gingivitis in snuff users in Sweden, a number of other studies found no relationship of gingivitis or gingival bleeding with the use of ST. Perhaps more indicative of an effect is the evidence relating ST use to gingival recession, attachment loss, dental caries and tooth loss. For all four endpoints, however, the evidence is somewhat inconsistent. From the data available, it is difficult to judge whether this variation is due to differences in type of product used, different definitions of endpoints, or failure to take into account other factors associated with poor dental health, including age, smoking, socio-economic status (education) and the number of visits to the dentist. At present the evidence on attachment loss, dental caries, and tooth loss must be regarded as no more than suggestive of an association with the use of ST.

The evidence relating to gingival recession seems rather stronger with four out of eight studies of snuff or unspecified ST reporting a significant association. There is huge variation in the reported prevalence between studies, marked heterogeneity in the strength of the reported associations in most of the studies reporting a relationship (the extremely high odds ratio in one study [29] being clearly inconsistent with the other findings), and failure to adjust for relevant potential confounding variables. However, given also that two of the three studies not finding an association were in children or adolescents where reported prevalence was very low indeed, and that the results are supported by an experimental study [18] showing a clearly lower risk in users of portion-bag than loose snuff, a true effect seems quite probable.

## Conclusion

Detailed assessment of the overall risks and benefits of ST use to the public health would require consideration of the whole spectrum of its possible health effects and is beyond the scope of this review. However, we do note that there are numerous reports, including our own publications on oral cancer [57] and on circulatory disease [56], which support the risks of smoking-related diseases from ST as being generally much less than those from smoking. This review confirms the strong relationship of oral mucosal lesions to ST use, shows that prevalence and severity is related to the type and amount of the product used, and that the lesion is reversible on quitting. The evidence relating other oral lesions to ST use is less clear. A causal relationship of snuff use with gingival recession seems probable, but not certain. The relationships between CT use and dental caries and between ST use and attachment loss are less clear, and the evidence here may be regarded only as suggestive of a causal relationship. There seems no real indication that ST use affects gingivitis (or gingival bleeding). Data are too limited to draw reliable conclusions for other endpoints, including oral pain.

## Competing interests

GK and RW work for Philip Morris International (PMI), R&D. PNL, founder of P.N. Lee Statistics and Computing Ltd., is an independent consultant in statistics and an advisor in the fields of epidemiology and toxicology to a number of tobacco, pharmaceutical, and chemical companies.

## Authors' contributions

GK and RW completed an unpublished review on this subject in 2006. PNL re-examined the literature and extensively revised the review into a form suitable for publication. All authors read and approved the final manuscript.

## Pre-publication history

The pre-publication history for this paper can be accessed here:


